# The Oral–Gut Microbiome Axis: Microbial Transit, Ectopic Colonization, Systemic Associations, Salivaomics, and Clinical Control Points

**DOI:** 10.3390/dj14100625

**Published:** 2026-10-01

**Authors:** Mark L. Cannon, John Peldyak, David J. Vigerust

**Affiliations:** 1 Department of Otolaryngology–Head and Neck Surgery, Northwestern University Feinberg School of Medicine, Chicago, IL 60614, USA; 2 Division of Dentistry, Ann & Robert H. Lurie Children’s Hospital of Chicago, Chicago, IL 60611, USA; 3 Independent Researcher, Mount Pleasant, MI 48858, USA; njjn@ispmgt.com; 4 Woody L. Hunt School of Dental Medicine, Texas Tech University Health Sciences Center, El Paso, TX 79905, USA; davidvigerust@spectrumsolution.com

**Keywords:** oral microbiome, gut microbiome, ectopic oral bacteria, gastric transit, salivaomics, xylitol, *Fusobacterium nucleatum*, *Porphyromonas gingivalis*, colorectal cancer, periodontal disease

## Abstract

The oral cavity is a continuous microbial source for the gastrointestinal tract. Adults swallow approximately 0.5–1.5 L of saliva each day, carrying a large and variable bacterial load. Gastric acidity removes most ingested organisms, but survival increases with food buffering, acid tolerance, mucin or biofilm protection, and proton-pump inhibitor use. Oral taxa are therefore repeatedly detected in stool, intestinal mucosa, and diseased tissues. The strongest mechanistic evidence concerns *Fusobacterium nucleatum* in colorectal cancer and *Porphyromonas gingivalis* in inflammatory and metabolic pathways, although detection alone does not prove stable colonization or causality. This narrative review summarizes microbial transit, key taxa, salivaomics, systemic associations, and practical oral control points. It also identifies clinical control points, including probiotics and polyols such as xylitol, as selective microbiome-modulating adjuncts at varying stages of evidence. The evidence suggests a strong clinical message: treat oral dysbiosis for established dental reasons, while paired oral and gut studies determine when downstream systemic benefits are achieved.

## 1. Introduction

The mouth is not an isolated ecosystem. Saliva, desquamated cells, food, and biofilm fragments carry oral microorganisms into the stomach with every swallow. Most are cleared, but repeated exposure creates many opportunities for transient passage and, in a susceptible host, ectopic residence.

This distinction matters because the gut microbiome helps regulate barrier integrity, immune tolerance, and metabolism. Oral pathobionts that reach the intestine may contribute adhesins, proteases, bacteriocins, and inflammatory products that alter these functions. The biological effect depends on dose, viability, strain, community context, host immunity, medication use, and exposure duration.

This review poses four practical questions: how large is the swallowed microbial burden, what survives gastric transit, which oral taxa are found downstream, and how should clinicians interpret the systemic associations? In addition, how can oral health clinicians influence or help create control points? Xylitol is a potentially useful oral control substance because of its long history in dental prevention, and optical-density studies show selective inhibition of several oral pathogens [1]. The application of oral probiotics would constitute another control mechanism; oral clinical studies, predominantly those evaluating *Limosilactobacillus reuteri* strains DSM 17938 and ATCC PTA 5289, have examined gingivitis, periodontitis as an adjunct to scaling and root planing, peri-implant mucositis and peri-implantitis, dental caries and salivary *Streptococcus mutans* inhibition, oral candidiasis, halitosis, orthodontic plaque, recurrent aphthous stomatitis, and postoperative wound healing [2].

## 2. Literature Search and Scope

This narrative review used targeted searches of PubMed/MEDLINE, Crossref, and publisher records through 31 August 2026. A focused update covering publications from January 2023 through August 2026 was performed to expand recent, independent coverage across human cohorts, strain-resolved studies, multi-omics, mechanistic experiments, and clinical interventions. Search terms covered the oral–gut axis, ectopic oral colonization, gastric survival, salivaomics, individual taxa, systemic outcomes, and xylitol. We prioritized human metagenomic, culture, tissue localization, longitudinal, and systematic review evidence. We utilized animal and in vitro studies when they addressed mechanisms not readily tested in humans. The evidence is synthesized qualitatively; this is not a registered systematic review, and no pooled effect estimate was calculated.

## 3. Quantitative Burden: How Many Bacteria Are Swallowed Each Day?

### 3.1. Oral Microbial Load

Saliva commonly contains 10^8^–10^9^ bacterial cells or colony-forming units per milliliter, while mature dental plaque can approach 10^11^ organisms per gram [3,4,5]. The Human Oral Microbiome Database includes more than 700 named taxa or phylotypes, and strain-level and gene-level diversity is much greater [6,7]. No single person carries every listed taxon; the composition varies by age, dentition, diet, disease, medication, and sampling site.

### 3.2. Daily Oral Exposure to the Gut

Healthy adults generally produce 0.5–1.5 L of saliva daily [8]. Salivary flow is lower at rest and rises during chewing, eating, and oral stimulation [9]. Multiplying daily volume by common salivary bacterial concentrations yields a plausible swallowed burden of approximately 10^11^–10^12^ organisms per day, with substantial individual variation.

This estimate describes exposure, not successful colonization. Many cells are dead or injured, and most viable organisms are removed by gastric acid, bile, intestinal flow, resident microbial competition, and host immunity. Even so, comparative metagenomics demonstrates measurable continuity between oral and intestinal communities, particularly in disease or after disruption of normal colonization resistance [10].

## 4. Gastric Transit: What Survives Stomach Acid?

### 4.1. The Gastric Barrier

Fasting gastric juice typically has a pH of 1.5–3.5 and serves as an effective microbial barrier. Survival increases when meals buffer acidity, liquid transit shortens exposure, mucin or biofilm matrices shield cells, organisms mount an acid tolerance response, or medication raises gastric pH [11,12,13,14,15,16,17]. These conditions select rather than simply admit bacteria: acid-tolerant streptococci and other protected organisms are more likely to pass than acid-sensitive planktonic cells.

### 4.2. Interpreting Survival Estimates

Table 1 summarizes how each condition is expected to shift bacterial survival during gastric transit and the evidence supporting that direction of effect.

Figure 1 illustrates this conceptual model of gastric selection, ecological context, and host susceptibility as joint determinants of clearance, transient passage, or ectopic residence.

## 5. Oral Bacteria Documented in the Gut: A Species-Level Review

Oral taxa have been reported in stool, intestinal biopsies, abscesses, tumor tissue, and experimental gut models (see Supplementary Table S1). Methods do not provide equal evidence: culture supports viability, strain-resolved sequencing supports source attribution, and repeated longitudinal recovery is stronger than a single 16S rRNA detection. Table 2 summarizes representative organisms; the associated evidence base is reviewed in references [18,19,20,21,22,23,24,25,26,27,28,29,30,31,32,33,34,35,36,37,38,39,40,41,42,43,44,45]. 

## 6. Key Organisms and Mechanisms

### 6.1. Oral Streptococci

*Streptococcus mutans* and *Streptococcus sobrinus* tolerate acid through proton-pumping and stress response systems that also favor survival during gastric passage [14,15]. Strains of *S. mutans* carrying collagen-binding proteins have been isolated from cardiovascular tissues, but systemic detection should not be equated with routine intestinal colonization [18,49]. Experimental studies show that oral bacteria can induce intestinal T-helper 1 inflammation under permissive conditions [50]. *Streptococcus salivarius* is usually a commensal, whereas the *S. anginosus* group is more often associated with abscesses and gastrointestinal tumors. This mixture of commensal and pathogenic behavior within a single genus is precisely where a selective microbiome-modulating agent could matter clinically. Xylitol and related polyols, including erythritol, produce dose-dependent, organism-specific inhibition of several of these streptococci in optical-density and biofilm assays, in contrast to the broad, non-selective killing produced by antiseptic rinses; this selectivity is the basis for treating polyol exposure as a modifiable clinical control point rather than a general antimicrobial strategy [1]. In addition, probiotics have been reported to modify the clinical level of mutans streptococci in saliva [2] (see Section 9).

Strain differences are central to interpretation. The presence of an oral species in stool does not show that every oral strain has the same capacity for adhesion, invasion, bacteriocin production, or persistence. Collagen binding and other virulence traits may enrich selected strains at ectopic sites [18,49,51]. Likewise, experimental induction of intestinal inflammation requires a susceptible ecological and immune setting [50]. Clinical studies should therefore move beyond genus-level relative abundance and include absolute quantity, viability, and strain-resolved virulence data.

### 6.2. Veillonella and Selenomonas

Veillonella species use lactate produced by streptococci and can function in both oral and intestinal communities. A strain of *Veillonella atypica* isolated from athletes increased lactate conversion to propionate in an experimental model, providing evidence that a normally oral-associated taxon can affect gut metabolism [29,52]. Selenomonas species are prominent subgingival anaerobes and have also been reported in intestinal datasets, but their independent contribution to gut disease remains uncertain [53,54].

### 6.3. Fusobacterium nucleatum

*Fusobacterium nucleatum* subsp. *animalis* (Fna C2) provides the clearest example of an oral-to-gut cancer link. Independent genomic studies found enrichment in colorectal carcinoma relative to matched normal tissue [31,32]. FadA-mediated binding to E-cadherin activates beta-catenin signaling, while Fap2 can engage TIGIT and reduce natural-killer and T-cell activity [33,34]. These mechanisms explain the repeated clinical association.

Strain-resolved work showed that Fusobacterium persists in primary colorectal tumors and matched metastases, and that experimental treatment reduced tumor-associated bacterial load and growth [55]. *F. nucleatum* can tolerate brief acidic exposure, especially within a protective matrix [56]. Polyol inhibition is measurable in vitro, although direct clinical prevention of colorectal disease has not been established [1].

### 6.4. Periodontal Pathogens and Other Oral Taxa

*Porphyromonas gingivalis* can alter gut community structure and barrier function in animal models, linking oral exposure with systemic inflammation and metabolic changes [36,37,38]. *Treponema denticola* supplies dentilisin, and *Tannerella forsythia* contributes adhesins and inflammatory signals; oral Treponema antigens have also been reported in brain tissue [43,44]. Prevotella, Actinomyces, Rothia, and Bifidobacterium include taxa shared across oral and intestinal datasets, but shared names do not always mean that the same strain or ecological function is present [24,25,28,40,41,56].

Periodontal pockets can contain a dense, continuously renewed burden of Gram-negative anaerobes. Swallowing therefore continues even when a single organism cannot be cultured from the gut. The periodontal community may act through repeated delivery of lipopolysaccharide, proteases, outer-membrane vesicles, and inflammatory host products. This community-level model is consistent with the keystone-pathogen concept and with experimental changes in gut barrier function after oral exposure to *P. gingivalis* [36,37,38,54]. Prevotella lineages occupy both compartments, but oral and intestinal strains can be genetically and functionally distinct [57]. Actinomyces may appear in abdominal infection after disruption of mucosal barriers, Rothia is reported in inflammatory bowel disease datasets, and *Bifidobacterium dentium* carries traits compatible with both oral acid tolerance and intestinal survival [25,28,45,46,47,58]. These observations identify research targets rather than a single shared pathogenic pathway. 

## 7. Salivaomics and Integrated Oral–Gut Diagnostics

### 7.1. Saliva as a Diagnostic Biofluid

Whole saliva contains microbial and host DNA, RNA, proteins, antibodies, enzymes, metabolites, lipids, inflammatory mediators, extracellular vesicles, and viable and nonviable microorganisms. Researchers can examine these components using microbiomics, proteomics, transcriptomics, genomics, metabolomics, lipidomics, and extracellular vesicle analysis [59,60,61,62,63,64,65,66]. Saliva is therefore both the major vehicle of oral microbial exposure to the gut and an easily accessible specimen for measuring that exposure.

SalivaDB catalogs thousands of reported biomarkers across many disease categories, but database presence is not equivalent to clinical validity [60]. The strongest use cases have a defined specimen protocol, analytical performance, clinical threshold, and an action linked to the result. Point-of-care testing should complement examination and imaging, not replace them [60,61,62,63,64,65,66].

### 7.2. Paired Oral–Gut Testing

An organism found in stool may represent intestinal residence, transient passage, or repeated oral inoculation. Stool testing alone cannot establish the source. Paired saliva and stool collection provides the upstream context, and strain-resolved metagenomics, quantitative PCR, culture, and virulence genotyping can determine whether the same organism is present in both compartments. Longitudinal sampling is essential and will be most informative, enabling personalized health care. A single positive result may reflect recent swallowing, whereas repeated recovery after oral treatment more strongly suggests persistence. Host response measurements can be added to microbial results, including salivary cytokines, active matrix metalloproteinases, immunoglobulins, metabolites, and extracellular vesicles, along with fecal inflammation and systemic markers. The key question is not merely whether a taxon is present, but whether it is viable, active, abundant, and linked to inflammation.

### 7.3. Clinical Use and Limitations

In dentistry, saliva can support caries and periodontal risk assessment, monitor peri-implant inflammation, and support research into fungal, viral, and oral cancer biomarkers. In patients with inflammatory bowel disease, colorectal neoplasia, metabolic liver disease, or unexplained dysbiosis, oral testing may identify a reservoir that would otherwise remain untreated. Each proposed panel should demonstrate a potential clinical benefit. However, as we collect more data and possibly share it in a national de-identified database, we may discover health and disease correlations we currently have no conceptual understanding of.

Collection time, fasting, hydration, salivary flow, pregnancy, medication, smoking, oral hygiene, recent dental treatment, and sample handling all affect salivary composition. A paired oral–gut protocol should standardize these variables, document antibiotic and proton-pump inhibitor use, and assess whether treatment alters both oral abundance and downstream findings. This transforms the oral–gut axis from a broad association into a testable clinical pathway.

Standardization is especially important when changes are expected after treatment. Studies should report collection time, stimulation status, specimen volume, flow rate, processing interval, storage temperature, assay platform, detection threshold, and any recent food or oral hygiene exposure. Controls should include periodontal status, caries activity, smoking, antibiotic use, proton-pump inhibitor use, diabetes, and gastrointestinal disease. Without these details, apparent oral–gut concordance may reflect collection or compositional bias rather than biological factors. Table 3 summarizes these salivaomics domains and their relevance to oral and combined oral–gut diagnostic use.

## 8. Clinical Implications

The sections below are organized by outcome, and it is important to keep the two related but distinct pathways separate. The oral–gut axis, the focus of this review, describes oral bacteria that transit the gastrointestinal tract by swallowing and act on gut tissue, gut microbial communities, or gut-derived signaling. The broader oral–systemic axis includes additional routes, most notably hematogenous dissemination during chewing, brushing, or periodontal inflammation, and direct inflammatory signaling from periodontal tissue, by which oral organisms or their products reach distant sites such as the heart, brain, joints, or placenta without necessarily passing through the gut. Cardiovascular disease, Alzheimer’s disease, rheumatoid arthritis, and pregnancy outcomes are discussed below because they are prominent in the oral–systemic literature and because gut-mediated and hematogenous mechanisms are sometimes proposed together for the same disease; each subsection specifies which pathway the cited evidence actually supports rather than treating oral–systemic association as automatic proof of oral–gut involvement. However, even if oral pathobionts transit solely hematogenously, oral healthcare interventions may still control their origin.

### 8.1. Gastrointestinal Cancer, Inflammation, and Liver Disease

The strongest clinical signal is for colorectal cancer. *Fusobacterium nucleatum* subsp. *animalis* is repeatedly enriched in colorectal tumors, can activate oncogenic and immune-evasion pathways, and may persist in metastatic disease [31,32,33,34,66,67,68]. Other oral-associated taxa, including members of the *S. anginosus* group and *Peptostreptococcus stomatis*, are present in tumor microbiomes, but their causal roles are less certain [24,25]. In inflammatory bowel disease, oral-associated Fusobacterium, Prevotella, Rothia, and Veillonella are often enriched, and experimental ectopic oral colonization can drive mucosal T-helper 1 responses [28,51].

The portal circulation also creates an oral–gut–liver pathway. Experimental oral administration of *P. gingivalis* alters the intestinal microbiome, impairs barrier function, and promotes systemic metabolic changes [37,38]. These findings support a plausible contribution to fatty liver and cirrhosis, but human tissue detection and treatment studies remain too heterogeneous to conclude that periodontal therapy prevents liver disease.

### 8.2. Cardiovascular and Metabolic Disease

Oral bacteria and their products can enter the circulation directly during chewing, brushing, or periodontal inflammation, and indirectly through the gut. Periodontal pathogens and *S. mutans* have been detected in cardiovascular specimens, and inflammatory, endothelial, and platelet mechanisms are well described [18,53,69]. Clinical consensus recognizes a consistent association between periodontitis and atherosclerotic cardiovascular disease, while emphasizing that periodontal treatment has not yet been shown to confer cardiovascular benefit [70].

The pediatric perspective is important and very topical. A recently published nationwide Danish cohort linked severe childhood caries and gingivitis with higher adult atherosclerotic cardiovascular disease incidence [71]. This does not establish that the microbiome alone caused the later outcome, because diet, social conditions, health behavior, and inflammation can occur together across the life course. It does show that childhood oral disease is a meaningful health marker and a reasonable target for early prevention.

### 8.3. Neurodegeneration and Mental Health

*Porphyromonas gingivalis* DNA and gingipains have been reported in Alzheimer’s disease (AD) brain tissue, and oral infection in mice produced neuroinflammation and amyloid-related changes [72]. Researchers have also detected oral Treponema in brain samples [44]. Observational studies link tooth loss and oral inflammatory burden to cognitive decline, but reverse causation is a major concern because neurodegeneration also impairs oral hygiene, diet, and access to care [73]. However, observational studies also demonstrate that periodontal disease precedes clinical cognitive decline and that the severity of periodontal disease correlates with progression of AD [74].

Periodontitis and depression share inflammatory markers, pain, functional limitations, and social consequences. A systematic review found mixed human evidence but a biologically plausible neuroimmune relationship [75]. Dental treatment is justified for oral health and quality of life; claims that it prevents depression or dementia require prospective trials.

The preventive implications should remain proportional to the evidence. Maintaining teeth, controlling pain and inflammation, and preserving mastication are clear clinical goals at every age. Whether those measures also reduce later neurodegenerative risk is an important question, but it should be tested rather than assumed.

### 8.4. Autoimmunity and Pregnancy

*P. gingivalis* can citrullinate proteins and has been studied as one potential link between periodontal inflammation and rheumatoid arthritis [76]. Periodontitis is more common in rheumatoid arthritis, but shared smoking, genetic, and inflammatory risks complicate causal interpretation. Periodontal care should be coordinated with medical treatment without presenting it as a substitute for rheumatologic therapy.

Periodontal inflammation is associated with preterm birth and low birth weight, and oral organisms have been identified in amniotic and placental specimens [77,78,79]. Maternal xylitol trials provide a separate and more direct preventive example: reducing maternal mutans streptococci delayed transmission and reduced childhood caries [80]. These findings support maternal dental care while leaving the magnitude of obstetric benefit to well-controlled intervention trials.

The first 1000 days are particularly important because feeding, maternal microbial exposure, eruption, antibiotic use, and household transmission shape the developing oral and gut communities. Maternal xylitol is a practical component of care, not a replacement for dietary counseling, professional prevention, or treatment of active maternal disease [80]. Longitudinal mother–child studies should measure both compartments and separate microbial transmission from shared diet and environment.

### 8.5. Oral Cancer and Pediatric Prevention

Oral squamous cell carcinoma has a distinct microbial community enriched for several periodontal taxa. Fusobacterium adhesins and *P. gingivalis* signaling provide plausible mechanisms, but the microbiome is one component of a multifactorial disease dominated by tobacco, alcohol, human papillomavirus, host susceptibility, and social determinants [81,82,83]. For children, the most defensible clinical response to oral–systemic evidence is also the simplest: prevent early caries and gingivitis, control diet, support salivary flow, and establish durable daily care before dysbiosis becomes chronic.

## 9. Xylitol as a Clinical Control Point

This review treats xylitol in depth because it has the most extensive evidence base among candidate oral–gut control points: decades of clinical caries prevention trials, defined delivery systems, and organism-specific in vitro data [1,80,84,85,86,87,88,89,90,91,92]. As a non-fermentable sugar alcohol that selectively restricts cariogenic and other oral pathobionts while sparing beneficial commensals, xylitol functions as a prebiotic; it is presented here as one working example of a modifiable clinical control point, not as the only microbiome-modulating option.

### 9.1. Mechanisms and Evidence

Xylitol is a five-carbon polyol that has been used in preventive dentistry since the Turku Sugar Studies [84]. In *Streptococcus mutans*, uptake through the phosphoenolpyruvate phosphotransferase system produces xylitol-5-phosphate, which cannot proceed through ordinary glycolysis. Repeated import, dephosphorylation, and export waste cellular energy, lengthen the lag phase, reduce acid production, and can lower adherence and glucan formation [85].

Optical-density studies extend this mechanism beyond a single laboratory strain. Xylitol and erythritol produced dose-dependent inhibition of *Streptococcus mutans* and *Streptococcus sobrinus* biofilm growth, and broader screening found organism-specific effects against *S. anginosus* and *Fusobacterium nucleatum* [1]. These are in vitro results. They support selection of dose and delivery method, but they do not, by themselves, prove a systemic clinical outcome.

Xylitol has also been studied in syngeneic mouse cancer models, including tumor growth and osmotic-pump experiments [85,86]. Those findings are biologically interesting but should remain separate from established caries prevention recommendations until dose, pharmacokinetics, mechanism, and human relevance are clarified.

### 9.2. Delivery and Clinical Use

Chewing gum has the longest clinical record because it combines polyol exposure with salivary stimulation. Maternal use reduced transmission of mutans streptococci and subsequent caries in children [80]. Lozenges are useful when chewing is unsuitable, and a large adult trial found a greater effect on root surface caries than on coronal surfaces [87]. Systematic review evidence supports a preventive role for xylitol-containing products, but the certainty and magnitude of benefit vary by product, dose, adherence, and baseline risk [88,89]. Delivery matters because short contact time, low total dose, and poor adherence can render an otherwise active ingredient ineffective. Gum and lozenges produce repeated oral exposure, while rinses provide broad distribution but shorter contact. Toothpaste can combine xylitol or erythritol with fluoride and remineralizing phosphate systems. Laboratory and in situ studies of low-fluoride formulations containing sodium trimetaphosphate report improved control of enamel demineralization [90,91]. These formulations should be evaluated as complete products rather than assuming that one ingredient’s effect transfers unchanged to every formulation.

Nasal irrigation exposes a different microbial compartment. A randomized pilot study found that xylitol irrigation was feasible for chronic rhinosinusitis [92]. Nasal delivery may reduce upper-airway exposure and swallowed secretions, but it should not be presented as proven treatment for gut dysbiosis. Across delivery systems, the practical choice depends on age, caries risk, salivary flow, periodontal status, tolerance, and the ability to use the product consistently. Figure 2 summarizes these practical control points across the delivery systems described above.

The combined use of xylitol with a probiotic strain in a single synbiotic product may offer an additional avenue for managing oral control points within the oral microbiome [93].

## 10. Discussion

The oral–gut axis is best understood as repeated exposure filtered by ecology. A large oral inoculum reaches the stomach every day, but only a fraction remains viable, and a still smaller fraction persists in the intestine. Disease, medication, diet, inflammation, and loss of colonization resistance can change each step.

Three biological states can overlap but require separate evidence. Transit describes an oral organism detected once, shortly after swallowing, with no evidence of survival beyond a single passage. Persistence describes repeated recovery of the same strain over days to weeks without confirmed in situ replication. True colonization requires evidence of in situ replication and community integration, typically shown by stable strain-level recovery across multiple time points together with functional or transcriptional activity in the gut environment. Most oral-taxon-in-stool findings in this literature establish transit or, at best, persistence; only *Fusobacterium nucleatum* subsp. *animalis* in colorectal cancer, and to a lesser extent *Porphyromonas gingivalis* in experimental models, approach the standard required for colonization. Mechanistically, transit is governed largely by gastric and biofilm survival (Section 4); persistence and colonization require additional adhesion, nutrient acquisition, and immune-evasion mechanisms that are organism-specific, including FadA-mediated E-cadherin binding and Fap2-mediated immune evasion for *F. nucleatum* [33,34], and gingipain-mediated barrier disruption and dysbiosis induction for *P. gingivalis* [36,37,38].

Evidence is strongest when the same strain is found in oral and downstream sites, remains detectable over time, expresses a relevant virulence mechanism, and changes disease in an experimental system. *Fusobacterium nucleatum* subsp. *animalis* in colorectal cancer approaches this standard [31,32,33,34,55], and strain-resolved metagenomics shows that a specific *F. nucleatum* subsp. *animalis* clade, rather than the species as a whole, dominates the tumor niche [35], reinforcing the strain-level rather than species-level framing used throughout this review. *Porphyromonas gingivalis* has strong experimental evidence for effects on intestinal barrier integrity, induction of dysbiosis, gut barrier permeability changes, enterobacterial translocation to the liver, and metabolic endotoxemia in animal models [32,34,35]; human tissue and clinical outcome data confirming these gut-specific mechanisms remain sparser than the animal evidence and systemic inflammation [36,37,38]. For many other taxa, current evidence demonstrates association or transit rather than stable colonization.

This distinction prevents two errors. First, a matching genus in saliva and stool is not proof that an oral strain colonized the gut. Second, failure to prove colonization does not make oral exposure irrelevant; repeated passage can still deliver inflammatory products and transient metabolic activity. Indeed, repeated oral pathobiont exposure may strongly influence the gut microbiome via short-term viability, bacteriocin actions, and metabolite production. Paired, longitudinal, strain-resolved sampling is the most direct way to distinguish these possibilities.

Independent colorectal cancer cohorts agree that *Fusobacterium nucleatum* is enriched in tumor tissue [31,32], but effect sizes and the specific subspecies or clade implicated vary by cohort, sequencing depth, and tissue-sampling method; strain-resolved analysis suggests that some of this variability reflects genuinely different *F. nucleatum* populations across cohorts rather than measurement noise alone [35]. *Porphyromonas gingivalis* gut axis findings are more consistent in animal models than in human observational data, where confounding by diet and periodontal status is difficult to separate fully from a direct microbial effect. Evidence type also differs sharply by claim: the *F. nucleatum*–colorectal cancer and *P. gingivalis*–gut barrier associations rest on a combination of human tissue metagenomics, culture, and animal experiments; most other rows in Table 2 rest on human observational 16S rRNA surveys alone, with little or no animal or clinical trial evidence; and the polyol inhibition data in Section 9 are presently in vitro, or, for xylitol specifically, supported by clinical caries trials that were not designed to test gut outcomes. Two recent reviews reach a broadly similar conclusion from different angles: periodontal and other oral taxa can influence gut and systemic disease through multiple, only partially overlapping mechanisms, and that influence is currently best established for a small number of taxa rather than for the oral microbiome as a whole [94,95].

Several limitations run throughout the literature. Relative-abundance sequencing can make one taxon appear increased when another taxon has declined, and 16S rRNA methods may not resolve closely related strains. Cross-sectional studies cannot distinguish cause from consequence. Periodontitis, caries, smoking, diet, age, medication, metabolic disease, and socioeconomic conditions are interrelated, so incomplete adjustment can inflate oral–systemic associations. Tissue contamination is another concern when collecting or processing low-biomass samples. Stronger studies need absolute quantification, negative controls, strain-level comparison, repeated sampling, and predefined clinical endpoints.

This limitation deserves particular emphasis because most of the taxon–gut associations summarized in Table 2 rest on exactly this kind of data: a compositional shift detected by 16S rRNA sequencing, without absolute quantification, strain resolution, or a demonstrated mechanism. A taxon reported as increased in one 16S rRNA survey may reflect a true increase, a decrease in a competing taxon that shifts relative proportions, differences in DNA extraction or primer choice, or cohort-specific diet and medication exposure rather than a biologically meaningful change. Metagenomic or culture-based confirmation is available for only a minority of the organisms listed in Table 2, and that caveat, more than any single study result, should temper how clinicians use the associations in that table.

Clinical care should begin with established priorities: control caries and periodontal inflammation, restore salivary function, reduce frequent exposure to fermentable carbohydrates, and use oral probiotics, xylitol, or other adjuncts according to individual risk. Salivaomics may improve measurement and follow-up when assays are validated. These actions are justified by oral outcomes even while systemic intervention trials are instituted. Xylitol illustrates this distinction with evidence. Its established benefit is caries prevention, supported by randomized trials and systematic review [80,82,87,88,89]; its potential oral–gut or other systemic effects are currently supported only by in vitro and animal data [1,85,86]. Oral probiotics have also been evaluated in clinical trials, including randomized clinical trials, meta-analyses, and systematic reviews [96]. Mechanical plaque control, restoration of diseased teeth, periodontal therapy, salivary management, and dietary change address established causes and outcomes. Xylitol, probiotics, and saliva-based monitoring are adjuncts selected for specific patients and purposes. Presenting adjuncts within this framework preserves clinical credibility and makes future oral–gut outcome trials easier to interpret.

A targeted update of the 2023–2026 literature reinforces both the plausibility and the limits of the oral–gut model. Large strain-resolved transmission studies demonstrate why source attribution requires population-level methods rather than species presence alone [97], while paired oral–stool cohorts indicate that even profound antibiotic-associated loss of gut colonization resistance does not automatically permit broad oral colonization [98]. Human colorectal cancer cohorts have detected *Porphyromonas gingivalis* in fecal and tumor samples and linked its presence to clinical outcomes [99]. Recent saliva–RNA fractionation work further shows that biomarker results depend strongly on which salivary compartment is analyzed [100]. Multi-compartment profiling in pregnancy linked periodontitis with coordinated plaque, saliva, serum, stool, and metabolite signatures [101], and mechanistic experiments demonstrated that *P. gingivalis* can worsen colitis through gut microbial metabolism and Th17/Treg imbalance [102]. A large colorectal cancer cohort likewise identified a network of *Parvimonas micra*, *Fusobacterium nucleatum*, *Peptostreptococcus stomatis*, and *Porphyromonas* species, supporting a community-level rather than single-organism interpretation [103].

Recent methodological and intervention studies also clarify what translation requires. Cross-cohort shotgun metagenomics identified a reproducible periodontitis signature across five countries, providing a stronger definition of the upstream oral source than a single-cohort 16S rRNA survey [104]. Population-resolved analysis of 9560 metagenomes showed that *Fusobacterium nucleatum* subpopulations differ by oral and intestinal niche and by disease phenotype, reinforcing the need to report subspecies or clade rather than genus alone [105]. In a randomized periodontal treatment trial, adjunctive *Limosilactobacillus reuteri* altered salivary and subgingival community composition but did not consistently add clinical benefit, illustrating why microbiome change and patient benefit must be evaluated as separate endpoints [106]. However, probiotic supplementation was limited to 21 days, limiting the study’s applicability to normal clinical practice, which often involves much longer probiotic use.

The next research step is not another unpaired cross-sectional survey. It is a prospective design that measures oral disease, saliva, stool, relevant tissues or biomarkers, medication, and diet, then tests whether treating the oral source alters downstream microbial and clinical outcomes. That design would show where the oral–gut axis is causal, where it is a marker, and where it is clinically modifiable.

Taken together, the oral–gut axis is better understood through clinical management of critical control points than through a single claim about the oral microbiome. Gastric transit, strain-level persistence of organisms such as *Fusobacterium nucleatum* subsp. *animalis* and *Porphyromonas gingivalis*, and modifiable exposures such as fermentable carbohydrate frequency with probiotic and polyol use each represent a point at which the axis can be measured and, in some cases, intervened upon. Establishing causality at each control point requires the paired, longitudinal, strain-resolved designs described above; until those data exist, clinicians can act with confidence only at the control points already supported by direct evidence, namely oral hygiene, diet, and established xylitol and probiotic indications, while treating gut and systemic outcome claims for individual taxa or polyols as testable hypotheses rather than settled clinical guidance. Distinguishing what is already modifiable in practice from what remains a research priority is, in our view, the central clinical message of the oral–gut axis for managing chronic disease going forward.

## 11. Conclusions

The mouth provides a continuous microbial inoculum to the gastrointestinal tract. Gastric acidity and ecological competition remove most organisms, but oral taxa repeatedly reach the intestine, and selected organisms have well-supported roles in downstream disease. *Fusobacterium nucleatum* subsp. *animalis* in colorectal cancer is the clearest example; *Porphyromonas gingivalis* and other periodontal pathogens have plausible inflammatory and metabolic effects with varying levels of human evidence. Paired saliva and gut testing can distinguish exposure from persistence. Preventing and treating oral dysbiosis remains sound dental care, while well-designed longitudinal trials determine the size of any systemic benefit. Framing gastric transit, strain-level organism persistence, and modifiable exposures such as oral polyol and probiotic use as critical control points, rather than treating the oral microbiome as a single undifferentiated risk factor, is the approach most likely to translate into practical management of oral–gut-related chronic disease.

## Figures and Tables

**Figure 1:**
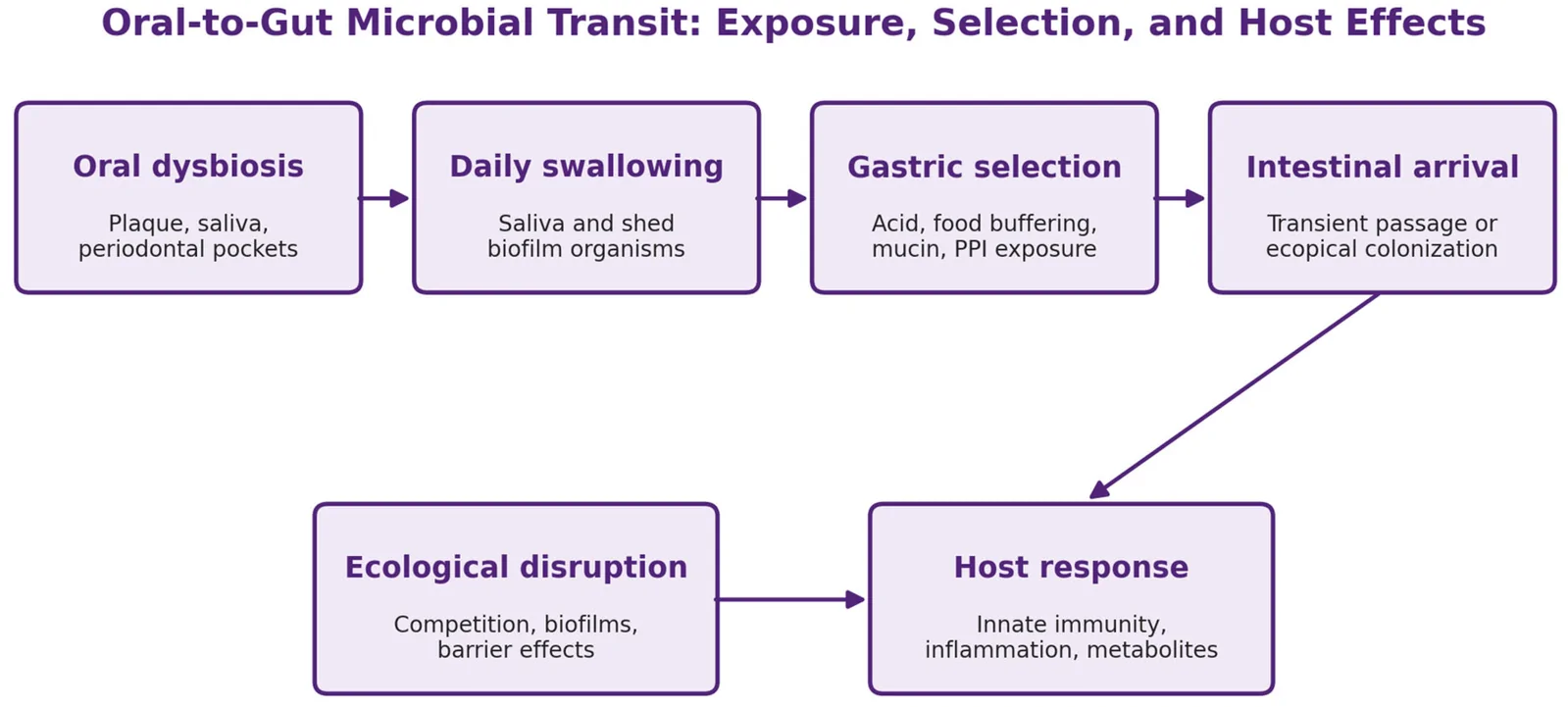
Conceptual model of oral microbial transit to the gastrointestinal tract. Gastric selection, ecological context, and host susceptibility determine clearance, transient passage, or ectopic residence.

**Figure 2:**
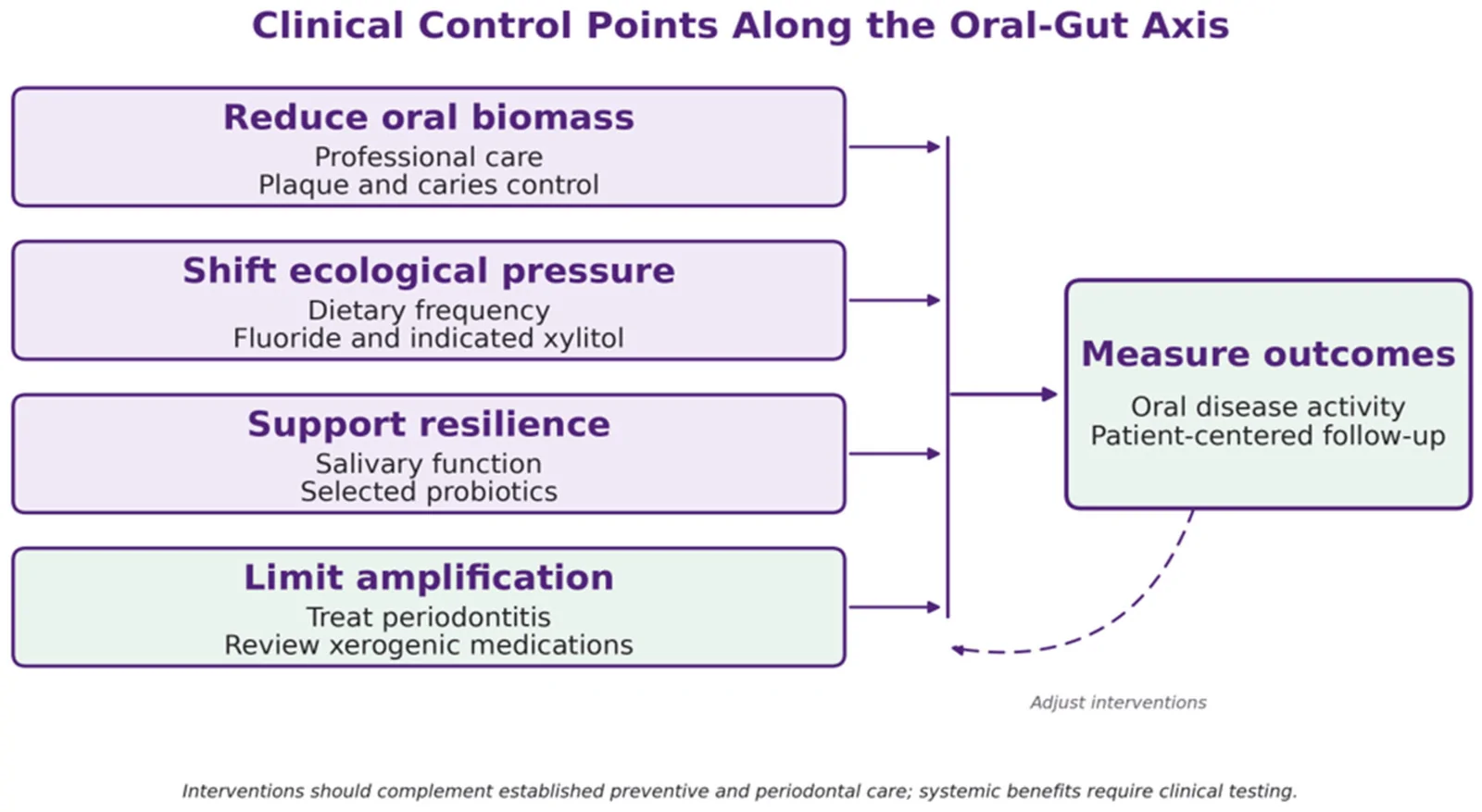
Practical control points for reducing dysbiotic oral microbial exposure. These measures complement established caries and periodontal prevention; systemic benefits require direct clinical evaluation.

**Table 1:** Factors that modify survival of swallowed oral bacteria during gastric transit.

Condition	Expected Effect and Rationale	Reference
Fasting acidity (pH 1.5–3.5)	Lowest survival because direct acid exposure kills most vegetative oral bacteria	Forsythe 2020 [11]
Meal buffering	Temporary increase as higher pH and food matrices reduce effective acid exposure	Sjögren et al. 2014 [12]
Mucin or biofilm protection	Potential increase because a surrounding matrix can reduce direct acid contact	Bansil and Turner 2006 [13]
Acid-tolerant organisms	Selective increase because stress responses improve survival during low-pH exposure	Quivey et al. 2000; Lemos and Burne 2008 [14,15]
Proton-pump inhibitor use	Sustained increase as higher gastric pH changes upper-GI and gut microbial exposure	Imhann et al. 2016 [17]

Published survival estimates vary because experiments differ in pH, exposure time, meal state, bacterial growth phase, and recovery method. The clinically relevant point is therefore not a single percentage. Instead, a very large inoculum is delivered repeatedly, and host or treatment conditions can shift the number and type of viable organisms reaching the small intestine.

**Table 2:** Oral bacterial species documented in the human gut. CRC = colorectal cancer; IBD = inflammatory bowel disease; NAFLD = non-alcoholic fatty liver disease; SCFA = short-chain fatty acid; CVD = cardiovascular disease.

Species/Taxon	Oral Role	Gut Detection Evidence	Clinical Significance in Gut
*Streptococcus mutans*	Primary caries pathogen	16S rRNA, culture (feces, intestinal biopsy)	Cardiovascular tissue detection in collagen-binding strains [18]; proposed intestinal bacteriocin effects on gut flora and linked to cerebral micro-bleeds.
*Streptococcus sobrinus*	Caries (high virulence)	16S rRNA (feces)	Co-occurrence with *S. mutans* in cariogenic oral biofilms; independent fecal or gut detection of *S. sobrinus* specifically has not been established in the reviewed literature [10].
*Streptococcus salivarius*	Commensal; probiotic strains	16S rRNA, culture (throughout GI tract)	Certain strains (e.g., K12, M18) are marketed oral/pharyngeal probiotics; direct evidence for sustained gastrointestinal colonization or anti-inflammatory activity specifically in the gut is limited within the reviewed literature [19,20].
*Streptococcus sanguinis*	Early oral colonizer	16S rRNA (feces, ileum)	Shown to aggravate colitis in an experimental model [21]; strongly associated with bacteremia and infective endocarditis [22,23].
*Streptococcus gordonii*	Dental plaque commensal	16S rRNA, metagenomics (feces)	Co-inoculation with a periodontal pathogen has been shown to alter gut microbial community composition in a murine model [24].
*Streptococcus anginosus*	Abscess-former	16S rRNA (colon biopsies)	Elevated in colorectal and esophageal cancer, alongside other members of the S. anginosus group [24,25].
*Streptococcus constellatus*	Abscess-former	16S rRNA (gut)	Appendicitis; abdominal abscesses [26].
*Lactobacillus salivarius*	Oral commensal	Culture, 16S rRNA (feces, colon)	Intestinal probiotic; reduces pathobionts [27].
*Veillonella parvula*	Dominant oral anaerobe	16S rRNA, metagenomics (feces, colon)	Detected in IBD-associated microbiome surveys [28].
*Veillonella atypica*	Oral commensal	Metagenomics (stool)	Gut transit survivor; lactate-to-propionate conversion and SCFA metabolism demonstrated in an experimental athlete-cohort model [29].
*Selenomonas sputigena*	Subgingival anaerobe	16S rRNA (feces, colon)	Detected via 16S rRNA in fecal surveys; established primarily as an oral pathobiont driving cariogenic biofilm virulence, with gut-specific clinical significance not yet determined [30].
*Fusobacterium nucleatum*	Oral anaerobe; periodontal	16S rRNA, culture, metagenomics (colon, CRC tissue)	Strongest oral species–CRC association; drives invasion and metastasis; strain-level work identifies a tumor-associated F. nucleatum subsp. animalis clade [31,32,33,34,35].
*Porphyromonas gingivalis*	Key periodontal pathogen	16S rRNA, culture (feces, colon, liver, brain)	NAFLD, IBD-associated dysbiosis, and Alzheimer’s disease brain tissue [36,37,38,39].
*Prevotella intermedia*	Periodontal pathogen	16S rRNA (feces, colon)	Reported in IBD- and gastric-cancer-associated microbiome surveys; Prevotella enrichment in oral and colonic samples from colorectal cancer patients has been reported [40]; a species-specific, mechanistic role for P. intermedia has been demonstrated in gastric cancer via a Perilipin-3-dependent pathway [41].
*Tannerella forsythia*	Red complex pathogen	16S rRNA (feces, colon)	Proposed role in colorectal carcinogenesis and gut inflammation as a red-complex pathogen [42].
*Treponema denticola*	Red complex pathogen	16S rRNA (feces, gut biopsies)	IBD-associated dysbiosis; produces dentilisin, a virulence protease [43], whose activity in the gut environment is biologically plausible but not directly demonstrated; oral Treponema antigens have been reported in Alzheimer’s disease brain tissue [44].
*Actinomyces israelii*	Oral actinomycete	Culture (abdominal disease)	Abdominal actinomycosis [45].
*Rothia dentocariosa*	Oral commensal	16S rRNA (feces)	Case reports link to bacteremia in inflammatory bowel disease patients receiving biologic therapy [46]
*Bifidobacterium dentium*	Oral-origin	Culture, 16S rRNA (colon)	Acid-tolerant gut colonizer [47].
*Eikenella corrodens*	Oral commensal	16S rRNA, culture (gut abscesses)	Intra-abdominal and liver abscesses [48].

Table 2 reflects varying strength of evidence across rows. Several entries rest on genus-level 16S rRNA community profiling rather than species-confirmed detection, and some clinical significance claims describe associations reported for a related species or for the genus as a whole rather than the named species itself. The strongest, most directly supported rows are *Fusobacterium nucleatum* and *Porphyromonas gingivalis*, consistent with Section 6 below.

**Table 3:** Salivaomics domains and their relevance to integrated oral–gut diagnosis.

Salivaomics Domain	Representative Analytes	Oral Diagnostic Relevance	Oral–Gut Diagnostic Relevance
Microbiomics	Bacterial, fungal, viral, and phage profiles; pathogen panels; dysbiosis indices	Caries, periodontal, peri-implant, fungal, and viral risk; treatment response	Comparison with stool or intestinal samples; source attribution; repeated oral seeding versus persistence
Proteomics and immunology	Cytokines, enzymes, immunoglobulins, matrix metalloproteinases, antimicrobial peptides	Inflammation, tissue breakdown, immune status, periodontal activity	Comparison with fecal and systemic inflammatory markers; barrier and host response assessment
Transcriptomics	mRNA, microRNA, circular RNA, long noncoding RNA	Oral cancer and inflammatory disease research; treatment response	Shared host response signatures and longitudinal disease monitoring
Genomics and epigenomics	Host DNA, microbial DNA, virulence genes, methylation, cell-free DNA	Host susceptibility, pathogen genotyping, precision risk assessment	Strain-resolved matching of oral and gut organisms; virulence and resistance tracking
Metabolomics and lipidomics	Lactate, nitrate/nitrite, short-chain fatty acids, amino acids, lipids, oxidative stress products	Diet–microbiome function, caries ecology, nitric oxide biology, inflammatory activity	Functional comparison of oral and gut microbial metabolism; metabolic and inflammatory phenotyping
Extracellular vesicles	Host exosomes and microbial vesicles carrying proteins, RNA, DNA, and metabolites	Cancer biology, inflammatory monitoring, intercellular signaling	Potential tracking of oral microbial and host signals across the gastrointestinal and systemic compartments

## Data Availability

No new data were created or analyzed in this study. Data sharing is not applicable to this article.

## Supplementary Materials

The following supporting information can be downloaded at: https://www.mdpi.com/article/10.3390/dj14100625/s1, Table S1: Oral Bacterial Taxa Reported in Intestinal, Fecal, or Gastrointestinal Samples.
