# Supplementary material for: The Oral–Gut Microbiome Axis: Microbial Transit, Ectopic Colonization, Systemic Associations, Salivaomics, and Clinical Control Points
Source: Dent J (Basel). 2026 Oct 1;14(10):625. doi: 10.3390/dj14100625 (PMC13703140; doi:10.3390/dj14100625)
Supplement: Supplementary file 1 [file dentistry-14-00625-s001.zip › dentistry-4523330-supplementary.pdf]

dentistry journal

MDPI

Supplementary Data

The Oral–Gut Microbiome Axis: Microbial Transit, Ectopic Colonization, Systemic Associations, and Clinical Control Point

Table S1. Oral Bacterial Taxa Reported in Intestinal, Fecal, or Gastrointestinal Samples

This condensed taxonomic table corresponds to the master list removed from the main manuscript. Detection in feces, intestinal contents, or tissue does not by itself establish durable colonization, directionality, or causation.

Entry	Phylum	Family	Taxon
1	Firmicutes	Streptococcaceae	*Streptococcus mutans*
2	Firmicutes	Streptococcaceae	*Streptococcus sobrinus*
3	Firmicutes	Streptococcaceae	*Streptococcus salivarius*
4	Firmicutes	Streptococcaceae	*Streptococcus sanguinis*
5	Firmicutes	Streptococcaceae	*Streptococcus gordonii*
6	Firmicutes	Streptococcaceae	*Streptococcus mitis*
7	Firmicutes	Streptococcaceae	*Streptococcus oralis*
8	Firmicutes	Streptococcaceae	*Streptococcus parasanguinis*
9	Firmicutes	Streptococcaceae	*Streptococcus infantis*
10	Firmicutes	Streptococcaceae	*Streptococcus cristatus*
11	Firmicutes	Streptococcaceae	*Streptococcus australis*
12	Firmicutes	Streptococcaceae	*Streptococcus anginosus*
13	Firmicutes	Streptococcaceae	*Streptococcus constellatus*
14	Firmicutes	Streptococcaceae	*Streptococcus intermedius*
15	Firmicutes	Streptococcaceae	*Streptococcus vestibularis*
16	Firmicutes	Lactobacillaceae	*Lactobacillus salivarius*
17	Firmicutes	Lactobacillaceae	*Lactobacillus rhamnosus*
18	Firmicutes	Lactobacillaceae	*Lactobacillus fermentum*
19	Firmicutes	Lactobacillaceae	*Lactobacillus gasseri*

Entry	Phylum	Family	Taxon
20	Firmicutes	Lactobacillaceae	*Lactobacillus casei*
21	Firmicutes	Lactobacillaceae	*Lactobacillus acidophilus*
22	Firmicutes	Lactobacillaceae	*Ligilactobacillus salivarius* (formerly *Lactobacillus salivarius*)
23	Firmicutes	Veillonellaceae / Selenomonadaceae	*Veillonella parvula*
24	Firmicutes	Veillonellaceae / Selenomonadaceae	*Veillonella atypica*
25	Firmicutes	Veillonellaceae / Selenomonadaceae	*Veillonella dispar*
26	Firmicutes	Veillonellaceae / Selenomonadaceae	*Veillonella rogosae*
27	Firmicutes	Veillonellaceae / Selenomonadaceae	*Selenomonas sputigena*
28	Firmicutes	Veillonellaceae / Selenomonadaceae	*Selenomonas noxia*
29	Firmicutes	Veillonellaceae / Selenomonadaceae	*Selenomonas infelix*
30	Firmicutes	Veillonellaceae / Selenomonadaceae	*Dialister invisus*
31	Firmicutes	Veillonellaceae / Selenomonadaceae	*Dialister pneumosintes*
32	Firmicutes	Veillonellaceae / Selenomonadaceae	*Megasphaera micronuciformis*
33	Firmicutes	Gemellaceae	*Gemella haemolysans*
34	Firmicutes	Gemellaceae	*Gemella morbillorum*
35	Firmicutes	Aerococcaceae	*Abiotrophia defectiva*
36	Firmicutes	Aerococcaceae	*Granulicatella adiacens*
37	Firmicutes	Aerococcaceae	*Granulicatella elegans*
38	Firmicutes	Peptostreptococcaceae	*Peptostreptococcus stomatis*
39	Firmicutes	Peptostreptococcaceae	*Parvimonas micra* (formerly Micromonas micros)
40	Firmicutes	Peptostreptococcaceae	*Peptoniphilus harei*
41	Actinobacteriota	Actinomycetaceae	*Actinomyces naeslundii*
42	Actinobacteriota	Actinomycetaceae	*Actinomyces odontolyticus*
43	Actinobacteriota	Actinomycetaceae	*Actinomyces israelii*
44	Actinobacteriota	Actinomycetaceae	*Actinomyces viscosus*
45	Actinobacteriota	Actinomycetaceae	*Actinomyces georgiae*
46	Actinobacteriota	Actinomycetaceae	*Actinomyces gerencseriae*

Entry	Phylum	Family	Taxon
47	Actinobacteriota	Rothiaceae	*Rothia dentocariosa*
48	Actinobacteriota	Rothiaceae	*Rothia mucilaginosa*
49	Actinobacteriota	Rothiaceae	*Rothia aeria*
50	Actinobacteriota	Bifidobacteriaceae	*Bifidobacterium dentium*
51	Actinobacteriota	Propionibacteriaceae	*Cutibacterium acnes* (formerly Propionibacterium acnes)
52	Bacteroidota	Porphyromonadaceae	*Porphyromonas gingivalis*
53	Bacteroidota	Porphyromonadaceae	*Porphyromonas endodontalis*
54	Bacteroidota	Porphyromonadaceae	*Porphyromonas somerae*
55	Bacteroidota	Prevotellaceae	*Prevotella intermedia*
56	Bacteroidota	Prevotellaceae	*Prevotella nigrescens*
57	Bacteroidota	Prevotellaceae	*Prevotella melaninogenica*
58	Bacteroidota	Prevotellaceae	*Prevotella copri* (oral-to-gut evolutionary lineage)
59	Bacteroidota	Prevotellaceae	*Prevotella pallens*
60	Bacteroidota	Prevotellaceae	*Prevotella denticola*
61	Bacteroidota	Prevotellaceae	*Prevotella oris*
62	Bacteroidota	Prevotellaceae	*Prevotella oulorum*
63	Bacteroidota	Prevotellaceae	*Prevotella salivae*
64	Bacteroidota	Bacteroidaceae	*Bacteroides fragilis*
65	Bacteroidota	Bacteroidaceae	*Bacteroides thetaiotaomicron* (oral/gut shared)
66	Bacteroidota	Tannerellaceae	*Tannerella forsythia*
67	Fusobacteriota		*Fusobacterium nucleatum*
68	Fusobacteriota		*Fusobacterium periodonticum*
69	Fusobacteriota		*Fusobacterium necrophorum*
70	Fusobacteriota		*Fusobacterium mortiferum*
71	Fusobacteriota		*Fusobacterium varium*
72	Proteobacteria		*Aggregatibacter actinomycetemcomitans*
73	Proteobacteria		*Haemophilus parainfluenzae*

Entry	Phylum	Family	Taxon
74	Proteobacteria		*Campylobacter rectus*
75	Proteobacteria		*Campylobacter gracilis*
76	Proteobacteria		*Campylobacter showae*
77	Proteobacteria		*Neisseria sicca*
78	Proteobacteria		*Neisseria mucosa*
79	Proteobacteria		*Eikenella corrodens*
80	Proteobacteria		*Desulfovibrio fairfieldensis* (oral detection; gut resident)
81	Spirochaetota		*Treponema denticola*
82	Spirochaetota		*Treponema lecithinolyticum*
83	Spirochaetota		*Treponema socranskii*
84	Synergistota		*Jonquetella anthropi*
85	Synergistota		*Pyramidobacter piscolens*
86	Synergistota		*Fretibacterium fastidiosum*
87	Saccharimonadota (Formerly Tm7)		*Saccharimonas aalborgensis* (oral ultra-small bacteria; gut detection)
88	Saccharimonadota (Formerly Tm7)		*TM7x (epibiont; metagenomically detected in gut)*
89	Chloroflexi		*Chloroflexi spp*. (oral phylotypes metagenomically detected at low gut abundance)
